# An Ultra-Long-Acting Dimeric Bictegravir Prodrug Defined by a Short Pharmacokinetic Tail

**DOI:** 10.21203/rs.3.rs-5959131/v1

**Published:** 2025-02-19

**Authors:** Benson Edagwa, Mohammad Ullah Nayan, Brady Sillman, Srijanee Das, Brandon Hanson, Ashrafi Sultana, Nam Thai Hoang Le, Suyash Deodhar, Alekha Dash, Samuel Cohen, Howard Gendelman

**Affiliations:** University of Nebraska Medical Center; University of Nebraska Medical Center; University of Nebraska Medical Center; University of Nebraska Medical Center; University of Nebraska Medical Center; University of Nebraska Medical Center; University of Nebraska Medical Center; University of Nebraska Medical Center; Creighton University; Department of Pathology and Microbiology, University of Nebraska Medical Center, Omaha, NE; University of Nebraska Medical Center

## Abstract

Ultra-long-acting (ULA) antiretroviral parenteral formulations, with low injection volumes, high resistance barriers, and short pharmacokinetic (PK) tails, can transform HIV-1 therapeutics. Here, we converted bictegravir (BIC), a potent daily oral antiretroviral drug, into monomeric and homodimeric ester prodrugs. The homodimeric prodrug nanosuspension, NMXBIC, shows sustained plasma BIC levels >16 times the protein-adjusted 95% inhibitory concentration (PA-IC_95_) for six months after a single injection in Sprague Dawley rats. The results paralleled a short PK tail with the potential for late dose forgiveness. The monomeric prodrug nanosuspension, NM2BIC, shows lower year-long plasma BIC concentrations above PA-IC_95_ after a single injection in Sprague Dawley rats. After repeated injections, NMXBIC and NM2BIC are well tolerated in New Zealand White rabbits. NMXBIC’s physicochemical properties and high BIC loading/unit mass of the prodrug contribute to its unique ULA PK profile. These results support its development as a ULA formulation for HIV-1 treatment and prevention.

## 1. Introduction

Despite significant advancements in antiretroviral therapy (ART), the fight against HIV-1 remains reliant on strict regimen adherence [1]. While daily oral ART is highly effective, suboptimal commitment leads to virological failure, transmission, and drug resistance [2]. Long-acting (LA) antiretroviral (ARV) formulations, like Cabenuva, combinations of cabotegravir (CAB) and rilpivirine (RPV), are a transformative alternative [3]. The Cabenuva LA regimen is as effective as daily oral ART when administered monthly or bimonthly. It also improves adherence and treatment outcomes in patients with a history of ART adherence challenges [4–7]. However, transitioning from daily to LA ART has introduced healthcare challenges. These include sustaining frequent clinic visits, laboratory testing, and patient confidentiality [8].

Other injectable ART obstacles include variable pharmacokinetic (PK) profiles and a prolonged subtherapeutic plasma drug concentration phase known as the PK tail [9, 10]. For instance, 22.5% of men and 63.4% of women, when given injectable CAB, experience a prolonged tail phase for up to a year after discontinuation [9]. Such extended PK tail increases the risk of drug resistance and breakthrough infection during treatment cessation [9, 11, 12]. In development, ultra LA (ULA) formulations focus now on optimizing dosing intervals, reducing injection volumes, and limiting PK tail durations. Synchronizing ULA antiretroviral therapy (ART) dosing with routine clinic visits could improve treatment outcomes and regimen continuation. The subcutaneous (SC) injection of lenacapavir (LEN, SUNLENCA) has already made progress. For treatment settings, SUNLENCA is given every six months in combination with daily oral ART in people living with HIV (PLWH) who are infected with multidrug-resistant HIV strains [13]. There is no other ULA small molecule agent that can be combined with SUNLENCA as part of a synchronous every six-month treatment regimen. This study reports a ULA homodimeric bictegravir (BIC) prodrug nanosuspension, NMXBIC, with six-month dosing and the potential to fill the niche [14]. Notably, BIC is a highly potent ARV drug with a high resistance barrier to viral mutation and is recommended as a first line of combination therapy [15].

A nanocrystalline prodrug approach produced NMXBIC from a BIC library of synthesized ester prodrugs. The new library consisted of monomeric BIC- myristate (MBIC), -stearate (M2BIC), -behenate (M3BIC), and a dimeric BIC -octadecanedioate (MXBIC). When administered to BALB/cJ mice and Sprague Dawley (SD) rats as single intramuscular (IM) injections, nanosuspensions of homodimeric MXBIC (NMXBIC) and monomeric M2BIC (NM2BIC) facilitated controlled injection site drug release. Notably, single IM injections of 45 and 90 mg/kg BIC equivalent doses of NMXBIC to SD rats provided a “plateau” phase of plasma BIC exposure, with BIC levels >16 times the protein-adjusted 95% inhibitory concentration (PA-IC_95_) for six months followed by a short PK tail. By comparison, a single IM dose of NM2BIC produced plasma BIC levels at or above the PA-IC_95_ for one year.

Alternative approaches to extended dosing frequencies and reduced PK tails include subcutaneous ARV-loaded solid and *in situ* forming implants [16–20]. While each one of them could broaden patient options, their uptake could be impacted by the need for surgical procedures for removable and non-tillable implants, limited drug loading and dose dumping for biodegradable implants, delayed drug absorption, PK variability, and adverse events from organic solvent based formulations [21–24]. Notably, histopathological examination of lymphoid, mucosal, and gut tissue showed no adverse reactions in SD rats treated with NMXBIC. SC and IM injections of NMXBIC administered to New Zealand White (NZW) rabbits produced well-tolerable injection site reactions (ISRs). More importantly, the strategy described in this work could potentially address prolonged PK tails of injectable ULA formulations. These data sets demonstrate the potential for a six-month aqueous nanosuspension of BIC with a short PK tail.

## 2. Results

### 2.1. Synthesis and characterization of monomeric and dimeric BIC ester prodrugs

**Figure 1:**
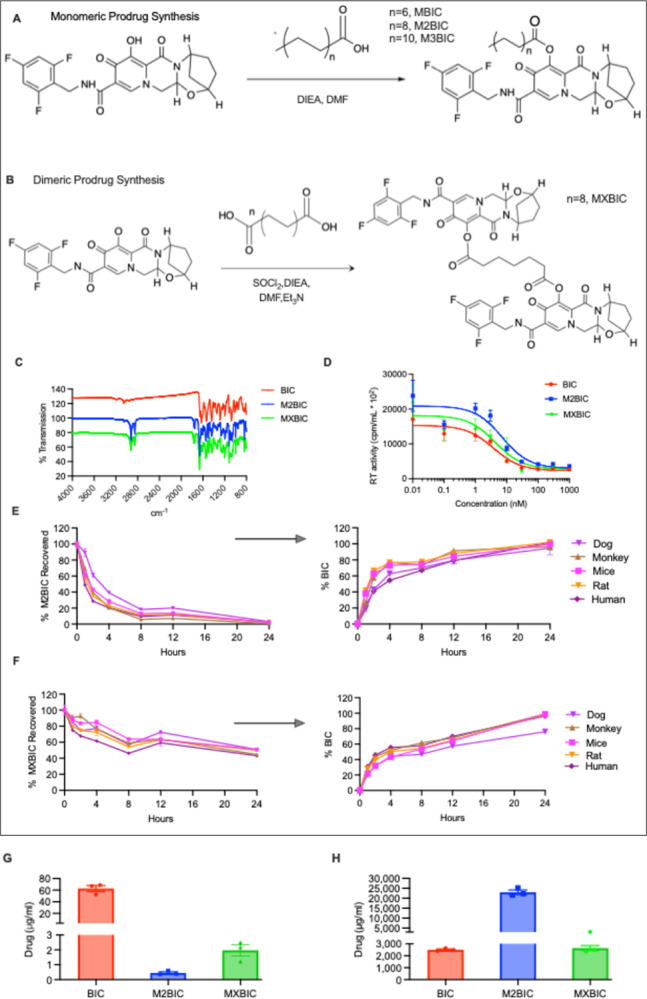
Synthesis and characterization of monomeric and dimeric BIC prodrugs. **A** BIC was chemically converted into monomeric ester prodrugs through one-step acylation reactions with either myristoyl (C-14), stearoyl (C-18), or behenoyl (C-22) chlorides. The formed prodrugs were designated MBIC, M2BIC, and M3BIC. **B** BIC octadecanedioate homodimer (MXBIC) was synthesized by conjugating two BIC molecules on each end of the optimal lipid. **C** An overlay of Fourier-transformed infrared (FT-IR) spectra for BIC (red), M2BIC (blue), and MXBIC (green), showing absorption bands at 2854 and 2918 cm^−1^ corresponding to the methylene asymmetric and symmetric stretching vibrations of the fatty acid derivatizing promoieties, respectively. The absorption band at 1751 cm^−1^ in the prodrug spectra represents the C=O stretch of the ester functional groups. **D** Antiretroviral half-maximal inhibitory concentration (IC_50_) of BIC (red), M2BIC (blue), and MXBIC (green) were evaluated over a concentration range of 0.1–1000 nM. This was determined by evaluating HIV-1 RT activity in culture fluids of HIV-1_ADA-_infected MDMs. The results are expressed as the mean ± SEM for n= 4 biological replicates. **E** and **F** Bioconversion of M2BIC and MXBIC solutions into parent BIC in dog, monkey, mice, rat, and human plasma. Plasma was incubated with a 1 μM solution of either M2BIC or MXBIC at 37°C, and prodrug conversion (left) into the parent BIC (right) was monitored over 24 hours. Results are expressed as mean ± SEM for sample numbers, n=3. **G, H** Aqueous and octanol solubility of BIC, M2BIC, and MXBIC. Results are expressed as mean ± SEM, for sample numbers, n=3, quantified using LC-MS/MS. DIEA, N, N-diisopropylethylamine; DMF, dimethylformamide; SOCl_2_, thionyl chloride; Et_3_N, triethylamine. **A-C** and **D-H** experiments were reproduced at least five and two times, respectively, with equvalent results.

Water-insoluble monomeric (MBIC, M2BIC, and M3BIC) and homodimeric (MXBIC) prodrugs of BIC were synthesized by esterification of the parent drug. The derivatization process employed naturally occurring fatty acids (Fig. 1A and B) with chemical yields of > 70%. The single-step chemical synthesis enables a scalable process. Their inherent solid forms facilitate their formulation into aqueous injectable nanosuspensions. The chemical structure and composition for each of the prodrugs made were affirmed by nuclear magnetic resonance (NMR), Fourier -transform infrared (FT-IR) spectroscopy, and electrospray ionization mass spectrometry (ESI-MS) (Fig. 1C and Supplementary Figs. 1–8). The recorded molecular ion peaks (M + H)+ at 660.33, 716.39, 772.45, and 1177.48 g/mol in the ESI-MS spectra identified the expected molecular weights for MBIC, M2BIC, M3BIC, and MXBIC, respectively (Supplementary Figs. 5–8). The unique X-ray powder diffraction (XRD) patterns for BIC, M2BIC, and MXBIC suggest a distinct arrangement of atoms within the crystal lattices (Supplementary Fig. 9A). For thermogravimetric analyses (TGA), a mass loss of less than 0.1% across a temperature range of 30°C to 300°C suggests that the final prodrug powders were anhydrous (Supplementary Fig. 10). Chemical modifications made to BIC were reversible for all the synthesized prodrugs. Hydrolysis studies demonstrated that solubilized forms of M2BIC and MXBIC were > 95 and 45% hydrolyzed within 24 hours after exposure to dog, monkey, mouse, rat, and human plasma, respectively (Fig. 1E–F). Additionally, M2BIC exhibited pH-dependent hydrolytic stability across a range of pH buffers. In contrast, MXBIC underwent uniform hydrolysis in different pH buffers, with up to half of the prodrug undergoing hydrolysis within 24 hours (Supplementary Fig. 11A and B). Efficient conversion of the prodrugs into parent BIC is also reflected by antiretroviral activity evaluation against HIV-1 reverse transcriptase (HIV-1 RT) activity in HIV-1_ADA_-challenged monocyte-derived macrophages (MDMs) (Fig. 1D). The 50% inhibitory concentration (IC_50_) of BIC, M2BIC, and MXBIC solutions were 3.75, 7.11, and 4.33 nM, respectively (Fig. 1D). MDMs exposed to M2BIC and MXBIC solutions also demonstrated comparable cell vitality as measured by the mitochondrial 3-(4,5-dimethylthiazol-2-yl)-2,5 diphenyl-tetrazolium bromide (MTT) assay (Supplementary Fig. 12). MTT determined cytotoxicity by evaluating cellular metabolic activity. The significantly reduced aqueous solubility of M2BIC and MXBIC facilitate chemical stability of the prodrugs within aqueous nanosuspensions. The aqueous solubilities of BIC, M2BIC, and MXBIC were 62, 0.45, and 1.96 μg/ml, respectively (Fig. 1G). Compared to M2BIC, BIC and MXBIC exhibited lower octanol solubilities (2499 and 2640 μg/ml for BIC and MXBIC, respectively vs 23.0 mg/ml for M2BIC) (Fig. 1H).

### 2.2. Formulation and characterization of aqueous BIC prodrug nanosuspensions

**Figure 2:**
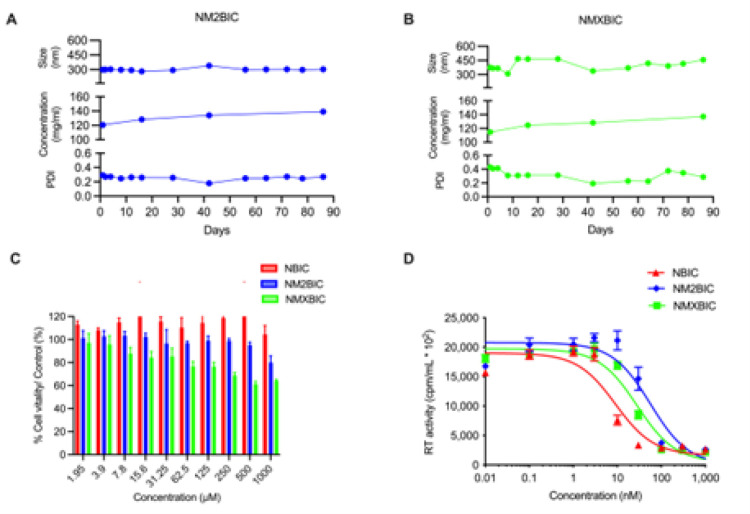
Characterization of BIC prodrugs nanosuspension. Particle sizes, polydispersity indices (PDIs), and prodrug concentrations of **A** NM2BIC and **B** NMXBIC were measured over 90 days at room temperature. Sizes and PDIs were determined by dynamic light scattering (DLS). Concentrations were measured using the UPLC- UV/Vis quantitation. Results are expressed as mean ± SEM, for n=3. **C** MTT determined Cell vitality after exposing MDMs to varying concentrations (1.95 – 1000 μM) of NBIC (red), NM2BIC (blue), or NMXBIC (green) for 24 hours. Results were normalized to untreated cells. Data is expressed as mean ± SEM, for n=4 biological replicates. **D** Antiretroviral half-maximal inhibitory concentration (IC_50_) values of NBIC (red), NM2BIC (blue), or NMXBIC (green) were evaluated in HIV-1 _ADA_ challenged-MDMs. Data was measured over a drug concentration of 0.1–1000 nM by HIV-1 RT activity in the culture fluids. Results are reported as mean ± SEM for n=4 biological replicates. **A-B** and **C-D** were experiments repeated at least five times and two times, respectively, generating equivalent results.

A scalable manufacturing process using microfluidization by high-pressure homogenization was developed to transform BIC prodrugs into surfactant-stabilized aqueous nanosuspensions [25]. The initial M2BIC and MXBIC nanosuspension, using poloxamer - 407 (P407) as the stabilizing surfactant, demonstrated encapsulation efficiencies of > 60%. The particle sizes of M2BIC nanosuspension (NM2BIC) and MXBIC (NMXBIC) were 300 nm (polydispersity index, PDI 0.29) and 375 nm (PDI 0.43), respectively (Fig. 2A and B). These formulations remained stable for three months at room temperature, maintaining consistent prodrug concentration and physicochemical properties, including color, viscosity, syringeability, particle size, and homogeneity (Fig. 2A and B). An optimal formulation with polysorbate 20 (Tween 20) and polyethylene glycol-3350 (PEG3350) stabilizers was designed, which significantly improved the drug concentration within the formulation from 120 mg/mL for the P407- containing formulations to 300–330 mg/mL (w/v) for the Tween 20/PEG 3350- containing formulation. The optimized NM2BIC and NMXBIC nanosuspensions were produced at encapsulation efficiencies of > 75%, particle sizes of 400–550 nm, and narrow PDIs (Supplementary Fig. 13). These formulations were stable in color, viscosity, particle sizes, and homogeneity for 142-days (Supplementary Fig. 13). XRD analyses confirmed the crystalline form of M2BIC and MXBIC within the formulation with distinct diffraction patterns from nanoformulated BIC (NBIC) (Supplementary Fig. 9B). Similar to unformulated M2BIC and MXBIC solutions, NM2BIC and NMXBIC nanosuspensions maintained cell vitality and cellular safety, underscoring the therapeutic potential (Fig. 2C). As expected for surfactant stabilized solid drug nanoparticles, nanoformulated forms of BIC (NBIC) and its prodrugs (NM2BIC and NMXBIC) exhibited modest increases in IC_50_ values compared to their solubilized forms. In particular, the IC_50_ values for NBIC, NM2BIC, and NMXBIC in MDMs were 9.04, 56.0, and 27.9 nM, respectively (Fig. 2D).

### 2.3. Macrophages facilitate drug absorption and secondary depots

**Figure 3:**
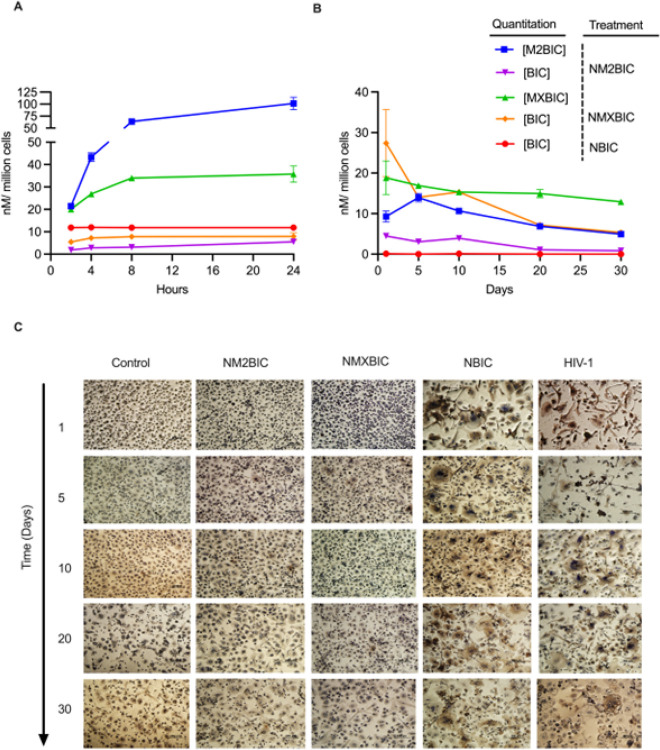
MDM drug uptake, retention and antiretroviral efficacy. **A** Uptake of BIC prodrug nanoformulations was measured in MDMs over 24 hours. This followed treatment with either NBIC, NM2BIC, or NMXBIC administered at 25 μM. **B** Drug retention and **C** antiretroviral efficacy in MDM were measured over 30 days. This followed an 8-hour treatment with either NBIC, NM2BIC, or NMXBIC at 25 μM. For uptake and retention studies, intracellular BIC and prodrug levels were quantified by UPLC UV-Vis. The results are expressed as mean ± SEM, for n=3 biological replicates. For tests of antiretroviral efficacy, MDMs were challenged with HIV-1 _ADA_ at a multiplicity of infection (MOI) of 0.1 infectious virions/cells. Infection was measured by cell-associated HIV-1p24 antigen immunostaining and HIV-1 RT activity in culture fluids. Representative images were taken at ×20 magnification. n=4 independent biological replicates. **A-C** experiments were repeated twice.

Macrophages facilitate absorption of nanoformulated drugs from the injection site. We previously demonstrated that macrophages infiltrate and uptake prodrug nanosuspensions at the injection site depot following IM injection [26]. Delivery of solid drug nanoparticles to macrophages enhances their half-life by facilitating intracellular and tissue drug storage [27]. We therefore examined drug uptake, retention, and antiretroviral activities in human MDMs after treating the cells with 25 μM of each nanoformulation. As shown in Fig. 3A and Supplementary Fig. 14A, BIC prodrug nanosuspensions facilitated enhanced intracellular drug delivery compared to NBIC. At 24 hours, intracellular prodrug levels were 61.9, 101, 200, and 35.7 nM/million cells for NMBIC (nanoformulated MBIC), NM2BIC, NM3BIC (nanoformulated M3BIC), and NMXBIC, respectively. To assess the prodrug’s intracellular conversion into the parent drug, BIC levels in the same samples were quantified. Intracellular BIC levels for NMBIC, NM2BIC, NM3BIC, NMXBIC, and NBIC-treated MDMs at 24 hours were 4.62, 5.50, 9.47, 7.95, 11.8 nM/million cells (Fig. 3A and Supplementary Fig. 14A). All the prodrug nanosuspensions, except NMBIC, facilitated sustained drug retention for up to 30 days following a single treatment (Fig. 3B and Supplementary Fig. 14B). On day 30, intracellular prodrug levels for NM2BIC, NM3BIC, and NMXBIC-treated MDM were 4.95, 42.4, and 12.9 nM/million cells, respectively (Fig. 3B and Supplementary Fig. 14B). No detectable drug was observed at 24 hours for NBIC, suggesting rapid drug release into culture media. Intracellular retention of BIC prodrugs paralleled improved antiretroviral activities. NM2BIC and NMXBIC protected against infection for up to 30 days after MDMs were treated for 8 hours with 25 μM of NM2BIC or NMXBIC, followed by an HIV-1_ADA_ challenge administered at 10-day intervals (Fig. 3C and Supplementary Fig. 15). The results demonstrate that BIC prodrugs improve intracellular drug delivery for sustained antiretroviral activities.

### 2.4. PK and biodistribution of nanoformulated monomeric prodrugs

**Figure 4:**
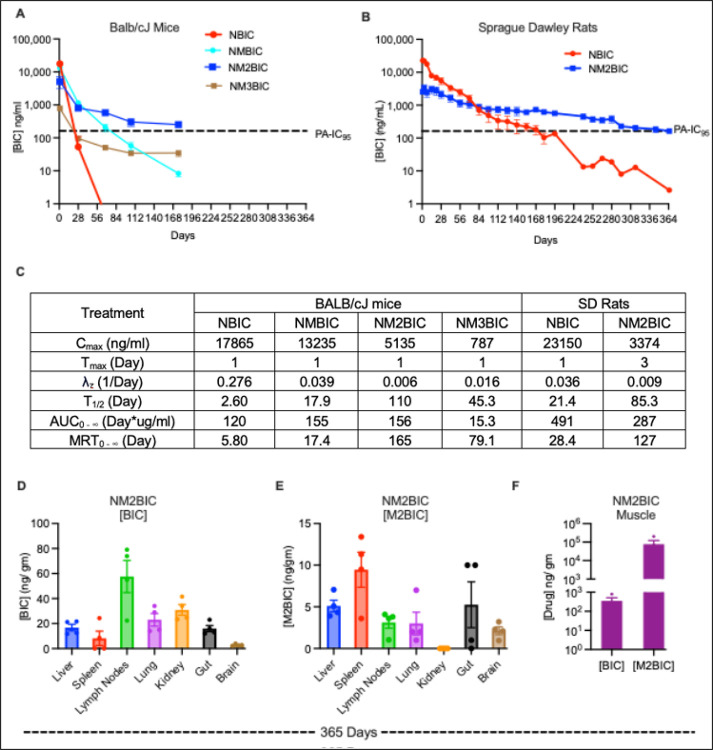
PK profiles for monomeric prodrug nanoformulations in BALB/cJ mice and Sprague Dawley rats. **A** Plasma BIC levels were measured after a single IM injection of either NBIC (red), NMBIC (cyan), NM2BIC (blue), or NM3BIC (brown) at a dose of 45 mg/kg BIC equivalent in the caudal thigh muscle of male BALB/cJ mice. For NM2BIC, the study was conducted over 365 days with n=3 animals. For all other groups, the study was conducted over 176 days with n=5 animals per group. **B** Plasma BIC levels after a single IM injection of either NBIC (red) or NM2BIC (blue) at a dose of 45 mg/kg BIC equivalent in the caudal thigh of Sprague Dawley rats were measured over 365 days. The study was initiated with n = 5 animals per group. However, a total of 3 animals in the NBIC group were lost during blood sampling on days 98, 126, and 194, respectively. One animal in the N2MBIC group was lost during blood sampling on day 168. The animals were sacrificed on day 365, and tissue drug levels were measured. **C** Plasma PK parameters for nanoformulated monomeric prodrugs and parent BIC were determined using non-compartmental analyses. C_max_, peak plasma concentration; T_max_, time required for C_max_, λ_Z_, individual estimate of the terminal elimination; T_1/2_, elimination half-life; AUC_0–∞_, area under the plasma concentration time curve (AUC) from 0 hours to infinity; MRT_0–∞_, mean residence time from 0 hours to infinity. Results are expressed as means except for the T_max_, which is expressed as median days. **D** Tissue BIC and **E** M2BIC levels for NM2BIC treated Sprague Dawley rats on day 365. **F** BIC and M2BIC levels at the injection site of NM2BIC-treated Sprague Dawley rats on day 365. Tissue drug levels for NBIC-treated Sprague Dawley rats on day 365 were below the limit of quantitation (LOQ= 1 ng/ml). All plasma and tissue drug levels were quantified by UPLC-MS/MS. Results are expressed as mean ± SEM. The horizontal dotted lines represent the protein-adjusted IC_95_ (PA-IC_95_ = 162 ng/ml).

The PK profiles of monomeric prodrug nanosuspensions (NMBIC, NM2BIC, and NM3BIC) were compared against nanoformulated BIC (NBIC) in male BALB/cJ mice after single IM injections at 45 mg/ kg body weight BIC-equivalents dose into the right caudal muscle. All the formulations had identical excipients (P407 stabilizer) and comparable particle sizes, homogeneity, and drug concentrations. On day 1, IM injection of NBIC and NMBIC demonstrated the highest peak plasma BIC concentrations compared to NM2BIC and NM3BIC (Fig. 4A). C_max_ values for NBIC and NMBIC were 17,865 ng/ml and 13,235 ng/ml, respectively, compared to 5,135 ng/ml and 787 ng/ml for NM2BIC and NM3BIC, respectively (Fig. 4C). Rapid drug dissolution and subsequent clearance from the muscle injection site for NBIC and NMBIC resulted in higher initial plasma BIC levels that rapidly fell below 4x PA-IC_95_ within 21- (305 ng/ml) and 68- days (207 ng/ml), respectively (Fig. 4A). On day 176, at sacrifice, BIC levels were below the limit of quantitation for NBIC treatment and slightly above the limit of quantitation for NMBIC treatment (Fig. 4A). Significantly lower BIC levels were recorded for NM3BIC treatment, where BIC levels fell below the PA-IC_95_ within two weeks, suggesting inefficient prodrug metabolism into BIC (Fig. 4A). Notably, NM2BIC showed improved PK profiles over NBIC, NMBIC, and NM3BIC by sustaining plasma BIC levels at or above PA-IC_95_ until day 295 (163 ng/ml) (Fig. 4A). On day 365, the mean plasma BIC concentration for NM2BIC treatment was 129 ng/ml (Fig. 4A). The mean residence time (MRT) for NM2BIC was 165 days compared to 5.80, 17.4, and 79.1 days for NBIC, NMBIC, and NM3BIC, respectively (Fig. 4C). NM3BIC exhibited a significantly lower plasma BIC decay rate (λ_z_ = 0.016 1/day) compared to NBIC and NMBIC (λ_z_ = 0.276 and 0.039 1/day), resulting in extended MRT of 79.1 days (Fig. 4C). The calculated half-life (T_1/2_) was 110 days for NM2BIC, compared to 2.60, 17.9 and 45.4 days for NBIC, NMBIC and NM3BIC (Fig. 4C).

NM2BIC and control NBIC formulations were further evaluated in SD rats administered single IM doses of 45 mg/kg BIC equivalent dose. Nanosuspensions with similar particle parameters were prepared for this PK study using Tween 20 and PEG3350 stabilizing surfactants. Similar to observations made in BALB/cJ mice, NM2BIC controlled the initial burst release of BIC more effectively than NBIC, resulting in a lower C_max_ value (3,374 ng/ml for NM2BIC vs. 23,150 ng/ml for NBIC) (Fig. 4B). Mean plasma BIC concentrations for NBIC were sustained above 4x PA-IC_95_ and 1x PA-IC_95_ for 84 days (700 ng/ml) and 168 days (183 ng/ml), respectively (Fig. 4B). However, plasma BIC levels among individual animals administered NBIC were highly variable. By comparison, plasma BIC concentrations for NM2BIC-treated animals were sustained at or above 4x PA-IC_95_ for at least four months (668 ng/ml on day 140) and above the PA-IC_95_ for one year (162 ng/ml on day 365). The median (minimum-maximum) plasma BIC concentration from day 7 to 140 was 1,409 (668–3,263) ng/ml (Fig. 4B). NM2BIC demonstrated sustained tissue drug levels. The average tissue BIC concentrations at 365 days after NM2BIC treatment were 57.6, 30.9, 23.0, 16.8, 16.0, 8.20, 2.90, and 356 ng/ gm tissue in the lymph nodes, kidney, lung, liver, gut, spleen, brain and injection site, respectively (Fig. 4D). Prodrug levels were significantly lower in all the tissues except the muscle injection site (77074 ng/gm of tissue) (Fig. 4E and F). These data confirmed that the muscle injection site was a primary drug depot. By comparison, BIC was not detected in any tissues following NBIC treatment. No differences in animal weights, organ-to-body weight ratio, and serum chemistry were recorded among NM2BIC-treated and age-matched control animals (Supplementary Fig. 16–18). Histopathological examination of hematoxylin and eosin (H&E)-stained tissue sections from NM2BIC-treated animals and age-matched controls, conducted by a board-certified pathologist, demonstrated normal liver, spleen, lung, kidney, and muscle tissue histology (Supplementary Fig. 19).

### 2.5. NMXBIC PK and tissue biodistribution

**Figure 5:**
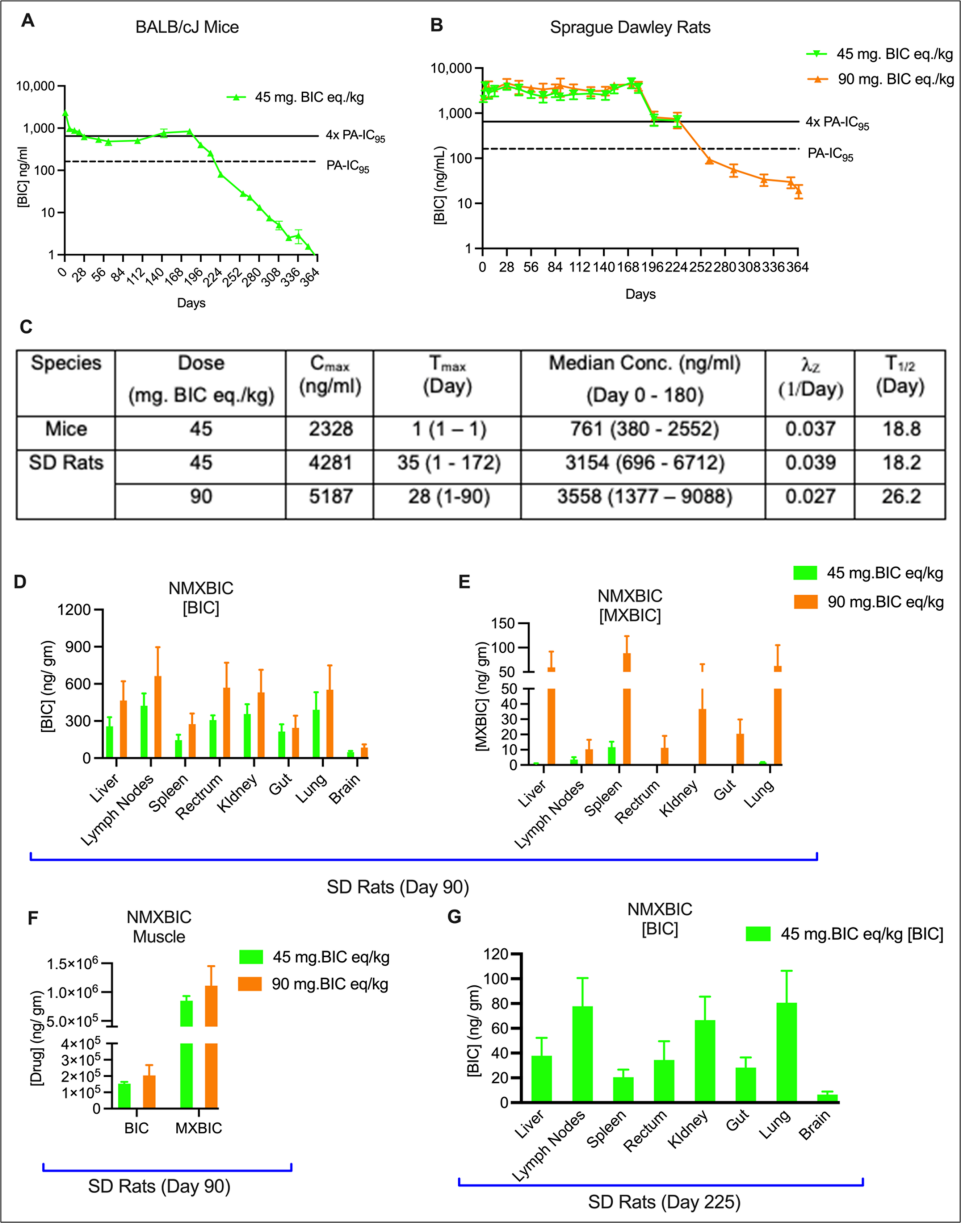
NMXBIC PK profiles in BALB/cJ mice and Sprague Dawley rats. **A** Plasma BIC concentrations were measured over 365 days after a single IM injection of 45 mg/kg BIC equivalent NMXBIC into the caudal thigh muscle of male BALB/cJ mice. Results are expressed as mean ± SEM, for n=3 animals. **B** Plasma BIC levels in Sprague Dawley rats following a single IM injection of NMXBIC at doses of 45 (green) and 90 (orange) mg/kg BIC equivalent in the caudal thigh muscle. Results are expressed as mean ± SEM for n=4 animals per group. The study was initiated with 8 animals per group, and blood sampling was alternated in 4 animals at each time point for 90 days. Half of the animals (four) from each group were sacrificed at 90 day, and the remainder in the 45 and 90 mg/kg BIC equivalent dose groups on days 225 and 365, respectively. The horizontal dotted and solid lines in **A** and **B** represent the BIC protein-adjusted IC_95_ (PA-IC_95_ = 162 ng/ml) and 4x of the PA-IC_95_ (648 ng/ml), respectively. **C** PK parameters for NMXBIC-treated animals were determined using non-compartmental analysis, C_max_, peak plasma concentration; T_max_, time required for C_max_, λ_Z_, individual estimate of the terminal elimination rate constant; T_½_, tail phase (post day 180) elimination half-life. All values are expressed as mean except T_max_, and median concentration from day 0 to day 180 are expressed as median (minimum-maximum). **D** Tissue BIC and **E** MXBIC levels on day 90 (n=4 animals per group) for both groups (green and orange for 45 and 90 mg/kg BIC equivalent dose group, respectively). F BIC and MXBIC levels at the injection site muscle on day 90 (green and orange for 45 and 90 mg/kg BIC equivalent dose group, respectively). **G** Tissue BIC levels on day 225 for 45 mg/kg BIC equivalent NMXBIC dose group. Tissue MXBIC levels were undetectable on day 225. **D-G** results are expressed as mean ± SEM for n=4 per animal groups. All the drug levels were quantified by UPLC-MS/MS.

NMXBIC PK was first tested in BALB/cJ mice, which differs significantly from NBIC or the monomeric prodrugs nanosuspensions. Plasma BIC levels for NMXBIC-treated mice exhibited a biphasic drug release profile compared to linear plasma BIC decay curves for the monomeric and parent drug formulations (Fig. 5A). Following a single IM injection of 45 mg/kg BIC equivalent dose, the initial peak plasma BIC level for NMXBIC-treatment were recorded within 24h post-injection, with a C_max_ value of 2,328 ng/ml (Fig. 5C). This is significantly lower than the C_max_ values for NM2BIC (5,135 ng/ml) and NBIC (17,865 ng/ml), suggesting a more controlled initial BIC release from NMXBIC. By day 7, mean plasma BIC levels declined to 970 ng/ml. However, beyond day 7, plasma BIC levels stabilized, fluctuating between 3x and 6x the PA-IC_95_ for six months (Fig. 5A). Notably, a rapid decline in plasma BIC levels was observed six months post-NMXBIC dosing, with a 29-fold decrease from day 180 to day 257 (28.0 ng/ml). By day 365, BIC levels dropped below 1 ng/ml. These data sets demonstrate the potential for NMXBIC to sustain efficacious BIC levels for six months with a short PK tail (Fig. 5A).

In a parallel study, NMXBIC’s PK profile was validated in SD rats administered a single IM injection of 45 and 90 mg/kg BIC equivalent dose. NMXBIC used for these studies consisted of Tween 20 and PEG3350 as the stabilizing surfactants. The differences in the shape of the PK profiles curve for NMXBIC and the monomeric ester prodrugs were also observed in SD rats. However, in rats with sufficient muscle mass for IM injections, NMXBIC produced a horizontal and consistently higher BIC concentration from day 1 to six months compared to linear plasma BIC decay curves for the monomeric and parent drug nanoformulations (Fig. 5B). Notably, there were no sharp fluctuations in plasma BIC concentrations in the first six months at both dose strengths (Fig. 5B). For the 45 mg/kg BIC equivalent dose of NMXBIC, median (minimum-maximum) plasma BIC concentration from day 1 to day 180 was 3154 (696–6712) ng/ml (Fig. 5C). Mean plasma BIC concentrations on days 28, 56, 84, 142, and 180 were 3951, 2688, 2798, 2541, and 3659 ng/ml. Despite the high plasma BIC concentrations in SD rats, the terminal decline in plasma BIC levels mirrored the short PK tail in mice, with drug concentrations declining to 682 ng/ml by day 225 (Fig. 5B). Doubling the dose in SD rats did not produce proportional plasma BIC exposure (Fig. 5B). The median (minimum-maximum) plasma BIC concentration from day 1 to 180 was 3558 (1377–9088) ng/ml (Fig. 5C). Mean plasma BIC concentrations on days 28, 56, 84, 142, and 180 were 4637, 3656, 3586, 3152, and 4034 ng/ml. Similarly, rapid plasma BIC decay was observed after day 180 (Fig. 5B). On day 365, the mean plasma BIC concentration was 19 ng/ml (Fig. 5B).

Tissue drug levels differed between the low and high-dose groups, with the higher dose yielding higher BIC and prodrug levels (Figs. 5D and E). For the high-dose group, BIC concentrations on day 90 were 668, 569, 553, 531, and 465 ng/gm tissue in lymph nodes, rectum, lung, kidney, and liver, respectively (Fig. 5D). By comparison, mean BIC levels on day 90 for the low-dose group were 422, 307, 391, 356, and 257 ng/ gm tissue in lymph nodes, rectum, lung, kidney, and liver, respectively (Fig. 5D). Tissue MXBIC levels in the low-dose group were 11.0, 1.00, and below the limit of quantitation in the spleen, lung, and liver, respectively (Fig. 5E). By comparison, the high-dose resulted in notably increased MXBIC tissue concentrations, particularly in the spleen, lung, and liver, with corresponding levels of 88.0, 62.0, and 59.0 ng/gm, respectively, on day 90 (Fig. 5E). Notably, both BIC and MXBIC were detected at the injection site on day 90 for both dose groups, supporting the formation of primary drug depots at the injection site (Fig. 5F). MXBIC concentrations at the injection site were 850558 and 1110100 ng/gm tissue for 45 and 90 mg/kg BIC equivalent dose group, respectively (Fig. 5F). BIC levels at the injection site muscle were 153577 and 204222 ng/ gm tissue for 45 and 90 mg/kg BIC equivalent dose group, respectively (Fig. 5F). Similar to the observed short PK tail after six months post-dosing, a significant decrease in tissue BIC and MXBIC levels were also recorded on day 225 for the 45 mg/kg BIC equivalent dose, suggesting efficient clearance of BIC and MXBIC from tissue reservoirs as well. The corresponding BIC levels on day 225 were 80.0, 77.0, 66.0, 37.0, and 34.0 ng/gm tissue in the lung, lymph node, kidney, liver, and rectum, respectively (Fig. 5G). No MXBIC was detected in tissues on day 225.

To evaluate the effect of injection volume and formulation concentration on NMXBIC absorption and PK profile, a less concentrated formulation (NMXBIC_low concent_ration) was made and injected intramuscularly to SD rats at a dose of 45 mg/kg BIC equivalent. NMXBIC_low concent_ration also demonstrated comparable PK profile curve that were observed with the high concentrated formulation (Supplementary Fig. 20A). However, notable differences were observed in the C_max_ and plasma BIC exposure. Median (minimum-maximum) plasma BIC concentrations from day 1 to day 180 for NMXBIC_low concentration_ was recorded at 1882 (470–3288) ng/ml with an average C_max_ of 2705 ng/ml (Supplementary Fig. 20B). Consistent with our previous observations, plasma BIC levels rapidly declined after six-months, affirming the short PK tail of NMXBIC (Supplementary Fig. 20A). The observed low BIC exposure from the dilute formulation could potentially be linked to the effect of drug-depot volume on drug absorption.

Histopathological examination of H& E-stained tissues treated with NMXBIC on day 365 found no morphological difference in all tissues compared with age-matched controls (Supplementary Fig. 21). There were no notable differences in animal body weights, organ-to-body-weight ratios, or comprehensive serum chemistry between the groups treated with NMXBIC and the age-matched control groups (Supplementary Figs. 22 and 23).

### 2.6. Injection site formulation tolerability

Injection site tolerability of NMXBIC and NM2BIC was comprehensively evaluated in New Zealand White (NZW) rabbits, a well-established model for depot injection studies due to their robust inflammatory and immune responses, as well as their suitability for testing human-equivalent doses [28]. Animals received IM or SC injections of NM2BIC or NMXBIC or formulation vehicle on day 1, followed by a second injection at a different site on day 26. On day 29, animals were sacrificed, and injection site tissues were collected for histological analysis. Notably, 1 ml IM injection of NMXBIC (150 mg) or NM2BIC (300 mg) on day 1 and day 26 were well tolerated and did not result in macroscopic alterations at the injection sites. Histological examination of H& E-stained injection site tissue sections on day 3 post-second injection revealed minimal myofiber necrosis in 1 of 6 and minimal to mild mixed cellular inflammatory reactions in 6 of 6 animals treated with NMXBIC (Table 1). Day 29 post-first injection analyses revealed moderate granulomatous inflammation and pseudocyst formation in 1 of 6 animals treated with NMXBIC (Table 1). Similarly, for NM2BIC, H&E-stained histological analysis on day 3 post-second injection indicated minimal myofiber necrosis in 1 of 6 animals and mild mixed cellular inflammation in 3 of 6 treated animals. By day 29 post-first injection, moderate granulomatous inflammation, and pseudocyst formation were noted in 2 of 6 animals treated with NM2BIC (Table 2).

2 ml SC injections of NMXBIC (300 mg) or NM2BIC (600 mg) or formulation vehicle were also well tolerated but caused thickening at the injection sites due to pseudocyst and pyogranulomatous inflammation. Minimal pyogranulomatous inflammation, mild mixed cellular inflammation, and pseudocyst presence was observed on day 3 post-second injection for NMXBIC treatment (Supplementary Table 1). Histopathological evaluation on day 29 post-first injection of NMXBIC indicated mild fibrosis, minimal to moderate mixed cellular inflammation, and pseudocyst development (Supplementary Table 1). Similar to NMXBIC, microscopic evaluation of day 3 after SC administration of NM2BIC revealed minimal to mild mixed cellular inflammation, isolated adipose necrosis, and pseudocyst formation (Supplementary Table 2). Histological analysis of tissue sections from the injection site on day 29 post-first injection predominantly revealed minimal to mild fibrosis within the subcutaneous layer, various degrees of cellular inflammation, minimal myofiber and adipose necrosis, and pseudocyst formation (Supplementary Table 2). Overall, the injection site findings for NMXBIC and NM2BIC represent the reported expected foreign body response to insoluble drug depots at the injection site [29, 30].

## 3. Discussion

BIC prodrug nanosuspensions manufacture, characterization, and PK profiles were evaluated. Lipophilic homodimeric and monomeric ester prodrugs with ideal physicochemical properties were made by esterifying BIC with biocompatible lipids. The two lead prodrug nanosuspensions, NMXBIC and NM2BIC, exhibited extended but distinct PK profiles. The extended BIC exposure was achieved through slow prodrug absorption, dissolution, and activation [26, 31]. Absorption and release of lipophilic prodrugs from aqueous-based nanosuspensions depend on several factors, including optimal prodrug lipophilicity, particle size, hydrolysis and lymphatic uptake [32]. For NMXBIC, these factors likely contributed to the observed sustained ‘plateau’ phase of plasma BIC exposure for up to 180 days. In SD rats, single IM doses of 45 and 90 mg/kg BIC equivalent NMXBIC produced median plasma BIC concentrations of 3154 and 3558 ng/ml, respectively, from day 1 to 180, followed by a short PK tail. These plasma BIC levels are 19 and 22-fold higher than the PA-IC_95_ and surpassed mean trough concentrations (C_tau_) of 2053 ng/ml observed in patients on the recommended 50 mg daily oral BIC dose [33]. Notably, NMXBIC improved BIC delivery to lymph nodes, rectum, lung, kidney, and liver. A single IM injection of NM2BIC resulted in a significantly slow rate of BIC decay. Here, plasma BIC levels were sustained at or above the PA-IC_95_ levels for up to one year with improved tissue drug delivery. The median plasma BIC concentration from day 7 to day 140 was 1409 ng/ml, exceeding the reported human C_tau_ levels for a 25 mg daily oral BIC dose (1052.3 ng/ml) [33]. It has been shown that once daily oral monotherapy with 25 and 50 mg of BIC for 10 days had comparable antiretroviral efficacy with a time-weighted average reduction in plasma HIV-1 RNA (log 10 copies/ml) of −1.33 (0.7) and − 1.37 (0.31), respectively [33]. Notably, the recorded higher prodrug to BIC ratio at the muscle injection sites for both NMXBIC and NM2BIC suggests the establishment of a primary prodrug depot at this site.

NM2BIC and NMXBIC were well tolerated in NZW rabbits, with limited injection site reactions and no evidence of muscle degeneration following IM administration of human-scale injection volumes. Signs of myofiber necrosis and mixed cell inflammation, which reflect an expected foreign body response that follows any IM injection, were also limited [34]. The observation of minimal to moderate levels of fibrosis, inflammatory granulomatous reactions, and pseudocyst formation was also consistent with the expected foreign body response to drug particles present at the injection site [35, 36]. Overall, both NM2BIC and NMXBIC were well-tolerated and support the likelihood of clinical safety. Moreover, the safety profiles of the solubilized and nanoformulated forms of MXBIC and M2BIC were demonstrated through cell vitality assays. The chosen lipids in prodrug chemical linkages are found in dietary sources like beef tallow and cocoa butter and have shown no adverse effects on serum LDL levels, supporting potential future prodrug formulation safety [37, 38]. Moreover, the safety profile of the surfactants used to stabilize the prodrug nanosuspensions is well-established [39].

This report is the first, to our knowledge, on PK profiling of ULA BIC formulations. While other groups have also reported LA BIC formulations, improvements in extending BIC’s half-life was quite limited. For instance, bilayer dissolving microneedles for intradermal delivery of BIC, demonstrated BIC plasma levels above PA-IC_95_ for only 4 weeks [40]. BIC and TAF-loaded poly (lactic-co-glycolic acid) nanoparticles have also been formulated using an oil-in-water emulsion approach, where BIC levels were sustained for only 30 days in mice [41]. The reported ULA NMXBIC could potentially facilitate the realization of complete ULA dual therapy for HIV treatment if combined with SUNLENCA. Both BIC and SUNLENCA demonstrate high resistance barriers, making them ideal candidates for HIV treatment [13, 42]. Efficacy data from ongoing clinical trials support dual combinations of SUNLENCA and BIC as initial and maintenance therapy for HIV treatment [43, 44]. Moreover, the short PK tail of NMXBIC could potentially be desirable in treating patients with adherence challenges as it will reduce the chances of drug resistance during missed doses and therapy discontinuations [45].

M2BIC and MXBIC chemical syntheses and formulation processes is scalable. Notably, nanosuspensions and prodrugs within the formulations are room-temperature stable for months, supporting their translational potential. Nanosized particle diameters enhance prodrug dissolution to produce a rapid onset of BIC upon parenteral administration. Comparable IC_50_ values of the solubilized forms of the prodrugs and native BIC in MDMs suggest efficient activation of the prodrugs through both enzymatic and chemical hydrolysis. The prodrug formulations demonstrated significantly improved intracellular BIC delivery and retention, potentially contributing to improved drug PK and biodistribution profiles [46].

Even though M2BIC and MXBIC feature a C-18 fatty acid linkage; the amount of BIC loaded on the prodrug linkage for each compound (one for M2BIC and two for MXBIC) significantly influences their physicochemical properties and hydrolysis profiles. M2BIC is 4.34-fold more hydrophobic than MXBIC. These solubility differences are likely influencing the dissolution and absorption profiles from the injection site depot. The observed “plateau” phase of plasma BIC exposure from NMXBIC treatment suggests controlled particle dissolution and simultaneous cleavage of the ester bonds to release two BIC molecules. The dimeric prodrug could also undergo a two-step hydrolysis process where one of the two ester bonds is slowly hydrolyzed to release one BIC molecule and a less hydrophobic monomeric BIC prodrug intermediate linked to a lipid with an ionizable carboxylate functional group. This intermediate is expected to undergo rapid hydrolysis to release the second BIC molecule. These hydrolysis mechanisms could potentially be contributing to the observed steady high plasma BIC exposure levels for NMXBIC. Beyond the six-month time point, all PK studies involving NMXBIC exhibit a more rapid decline in plasma BIC levels. This results in a shorter terminal PK tail, where the tail phase T_1/2_ ranges from 18 to 26 days. MXBIC loading in the formulation and injection volume significantly impacts the dissolution and absorption properties of NMXBIC, reflected by the PK profile of NMXBIC_low concentration_ in SD rats. Decreasing MXBIC concentration within the nanosuspension by 1.46-fold decreased median plasma BIC levels by up to 1.66-fold in SD rats. In contrast, a slow but steady decline in plasma BIC concentration observed with NM2BIC suggests slow prodrug absorption from injection site depot, given the lipophilic nature of the molecule.

The influence of a prodrug’s aqueous solubility on plasma BIC exposure was observed in the PK profiles of each compound. Specifically, native BIC displayed the highest aqueous solubility, resulting in rapid dissolution and absorption and subsequent, uncontrolled drug release on day 1. This rapid release did not favor the formation of a long-lasting depot for NBIC in mice. The C-14 modified monomeric BIC prodrug formulation, NMBIC, mirrored the burst release profile seen with NBIC (17679 ng/ml for NBIC compared to 13235 ng/m for NMBIC), resulting in rapid depletion of the drug depot. By comparison, enhancing prodrug lipophilicity through a BIC stearate prodrug formulation (NM2BIC), lowered peak plasma active drug concentrations and the rate of decay, suggesting the formation of a prolonged drug depot at the injection site. Further enhancement in prodrug lipophilicity through C-22 fatty ester BIC prodrug in NM3BIC significantly reduced prodrug dissolution and hydrolysis rates; diminishing plasma BIC concentrations.

In summary, two ULA BIC prodrug nanosuspensions, NM2BIC monomer, and NMXBIC homodimer, were created as potential next-generation HIV-1 treatment options. Mice and rats administered a single IM dose of NMXBIC rapidly achieved and sustained clinically relevant BIC concentrations for six months, followed by a short PK tail. Repeated SC and IM injections of NM2BIC and NMXBIC were well tolerated in NZW rabbits, supporting their continued evaluation as potential every six-months therapies.

## 4. Methods and Materials

### 4.1. Reagents

Reagents were obtained from commercial sources and used directly; exceptions are noted. BIC was purchased from BOC Sciences (Shirley, NY, USA). Myristoyl chloride, stearoyl chloride, behenic acid, octadecanedioic acid, pyridine, dimethylformamide (DMF), N,N-diisopropylethylamine (DIEA), polysorbate 20 (Tween 20), polyethylene glycol-3350 (PEG-3350), Pluronic F127 (poloxamer 407; P407), ciprofloxacin, 3-(4,5-dimethylthiazol-2-yl)-2,5-diphenyltetrazolium bromide (MTT), dimethyl sulfoxide (DMSO), 1-octanol, paraformaldehyde (PFA), and 3,3’-diaminobenzidine (DAB), were purchased from Sigma-Aldrich (St. Louis, MO, USA). Diethyl ether, acetonitrile (ACN), methanol, optima-grade water, ethyl acetate, hexanes, dichloromethane (DCM), Dulbecco’s Modified Eagle’s Medium (DMEM), phosphate-buffered saline (PBS), gentamicin, L-glutamine, potassium phosphate monobasic (KH_2_PO_4_), bovine serum albumin (BSA), and Triton X-100 were purchased from Thermo Fisher Scientific/Gibco (Waltham, MA, USA). Cell culture grade water (endotoxin-free) was purchased from Cytiva (Logan, UT, USA). Heat-inactivated pooled human serum was purchased from Innovative Biologics (Herndon, VA, USA).

### 4.2. Synthesis and characterization of BIC prodrugs

We synthesized three lipophilic monomeric prodrugs of BIC through single-step esterification reactions, each using linear biocompatible fatty acid chlorides of varying carbon chain lengths (C-14, C-18, and C-22), coined as MBIC, M2BIC, and M3BIC, respectively. One equivalent of BIC was first dried from anhydrous pyridine and then solubilized in anhydrous dimethylformamide (DMF) solvent under an argon atmosphere. The solution was then cooled to 4°C, followed by the addition of 2 equivalents of diisopropylethylamine (DIEA) base. Subsequently, 1.5 equivalents of the acyl chlorides were added, and the reactions were continued for 18 hours at 45°C. Upon completion of the reactions, the solvents were evaporated on a rotary evaporator, followed by dilution of the reaction mixture in dichloromethane (DCM) and successive extraction with 0.1 N HCl, NaHCO_3_, and brine solution. The DCM layer was dried over Na_2_SO_4_ and then concentrated using a rotary evaporator. The desired products were isolated by flash chromatography eluting with a 4:1 mixture of ethyl acetate and hexane. The desired compound fractions were collected, dried on a rotary evaporator, and then precipitated from hexane to obtain off-white powders. The prodrug powders were further dried under vacuum, producing average chemical yields of 70–75%.

Additionally, one dimeric BIC prodrug, MXBIC, was synthesized by activating octadecanedioic acid using thionyl chloride (SOCl_2_), followed by conjugation to BIC. Briefly, octadecanedioic acid (0.5 equivalent) was dried from benzene and reacted with ten equivalents of SOCl_2_ for 4 hours at 75°C. The reaction mixture was then dried by co-evaporating with 20 ml of benzene on a rotary evaporator. The dried sample was resuspended in 20 ml of anhydrous DMF under an argon atmosphere and cooled to 4°C. Four equivalents of DIEA and one equivalent of BIC were added to the reaction mixture and warmed to 45°C. After 16 hours, the reaction mixture was concentrated using a rotary evaporator and subjected to acid-base extraction as described for the monomeric prodrugs. The DCM layer was dried over Na_2_SO_4_ and then concentrated using a rotary evaporator. The concentrated sample was then purified by flash column chromatography eluting with a 4:1 mixture of ethyl acetate and ether. The desired compound fractions were collected and dried on a rotary evaporator to produce colorless foams. The dried compound was then lyophilized from THF and water, producing off-white powders with average chemical yields of > 70%.

All chemical reactions were conducted under an argon atmosphere unless otherwise specified. The final products were characterized by proton and carbon NMR (^1^H and ^13^C NMR) spectroscopy on a Bruker Advanced-III HD (Billerica, MA, USA) operating at 400 MHz, a magnetic field strength of 11.7 T. Distinct functional groups within the synthesized compounds was corroborated by FT-IR spectroscopy, performed on a PerkinElmer universal attenuated total reflectance Spectrum Two (Waltham, MA, USA). The molecular masses of the purified compounds were confirmed on an Exploris 480 mass spectrometer. Comparative crystallographic analyses of the synthesized compounds were conducted via powder X-ray diffraction (XRD) within a 2ø range of 2–50°, utilizing a PANalytical Empyrean diffractometer (PANalytical Inc., Westborough, MA, USA) equipped with Cu-Ka radiation (1.5418 A) at settings of 40 kV and 45 mA.

### 4.3. Aqueous and octanol solubility of BIC prodrugs

An excess amount of each compound was continuously mixed in either water or 1-octanol at room temperature for 24 hours. After saturation, the sample mixture was centrifuged at 20,000 × g for 10 minutes to separate the insoluble drug from the supernatant. The 1-octanol supernatant solutions were directly analyzed for drug content by LC-MS/MS. Aqueous supernatant solutions were lyophilized prior to reconstituting in methanol and drug quantitation by LC-MS/MS.

### 4.4. Solubilized prodrugs hydrolysis in plasma and different pH buffer solution

To investigate the role of enzymatic and chemical hydrolysis in BIC prodrugs activation into the parent drug, 1 μM solution of M2BIC and MXBIC in methanol (1% v/v) was incubated in plasma obtained from various species (dog, monkey, mice, rat, and human) or in buffer solutions of different pH values (2.0, 6.0, 8.0, and 10.6). BIC and prodrug levels in each sample were determined at pre-specified time points. Briefly, plasma or pH solution was preincubated at 37° C for 5 minutes. Then, 10 μl of a 100 μM solution of M2BIC or MXBIC in methanol was incubated in 990 μl of plasma or pH solution at 37°C for 24 hours, yielding a final prodrug concentration of 1 μM. At predetermined time intervals, 25 μl of sample was drawn from the mixture and mixed with 475 μl of 80% methanol in water containing 0.1% formic acid (Optima-grade). The sample mixture was vortexed for 3 minutes and centrifugated at 16,000 × g for 10 minutes. The resulting supernatants were collected and analyzed for drug content by UPLC-MS/MS using the quantitation method outlined in the supplementary section. Control samples were prepared following the same protocol, however, M2BIC and MXBIC solutions were added to plasma containing methanol. The pH solutions investigated in this experiment included 7.5 mM ammonium acetate (pH 6.0, adjusted with acetic acid), 7.5 mM ammonium bicarbonate (pH 8.0, adjusted with acetic acid), as well as pH-adjusted solutions involving 0.1% formic acid (pH 2.0) and 0.1% ammonium hydroxide (pH 10.6).

### 4.5. Nanoformulation preparation and characterization

BIC and each prodrug were formulated as aqueous nanosuspensions stabilized by non-ionic surfactants used in other parenteral products. Briefly, the water-insoluble prodrug powders were either dispersed in solutions of surfactants in phosphate-buffered saline (PBS) (11.9 mM potassium phosphate monobasic, 137 mM sodium chloride, 2.7 mM potassium chloride) or endotoxin-free water (pH 7.0), followed by microfluidization of the presuspension on an Avestin EmulsiFlex-C3 high-pressure homogenizer (Ottawa, ON, Canada) at 20,000 ± 1000 PSI until the desired nanoparticle size was achieved. Each formulation was assessed for stability (hydrodynamic particle diameter (size), polydispersity indices (PDIs), and zeta potential, appearance, and drug concentration (prodrug stability within the formulation) at room temperature. Size, PDI, and zeta potential were determined by dynamic light scattering (DLS) using a Malvern Zetasizer Nano-ZS (Worcestershire, UK). The stabilities of the nanosuspensions were monitored at room temperature. Compound (BIC or prodrugs) concentration in each nanosuspension was determined by diluting the formulations in methanol (1,000–10,000-fold dilution) followed drug quantitation by UPLC–ultraviolet/visible (UV/vis) spectrometry. The encapsulation efficiency was calculated using the equation: encapsulation efficiency (%) = (weight of drug in formulation/initial weight of drug added) × 100. Endotoxin concentrations in the formulations were determined by using a Charles River Endosafe nexgen-PTS system (Charles River, USA). All the preclinical formulations were readily syringeable using a 28 G needle.

The initial formulation screening for NBIC, NM2BIC, NMXBIC, NMBIC, and NM3BIC were produced in endotoxin-free water, using poloxamer P407 as the surfactant. For SD rats, optimal formulations were prepared in polysorbate 20 (Tween 20) and polyethylene glycol-3350 (PEG 3350) solutions in PBS. All the formulations used in SD rats PK studies had particle sizes of 300–550 nm and PDIs of 0.3 to 0.4.

### 4.6. PK and BD studies of monomeric prodrugs nanosuspensions

For mice PK studies, male BALB/cJ mice (weighing 26–30 gm, aged 6–8 weeks, obtained from Jackson Labs, Bar Harbor, ME, USA) were administered a single IM injection of either NBIC, NMBIC, NM2BIC, or NM3BIC at a dose of 45 mg/kg BIC equivalent. NBIC, NMBIC, and NM3BIC groups had five mice per group, and NM2BIC group had three mice. Blood samples were collected on days 1, 3, 7, 14, 21, 28, and then every two weeks post day 28. On day 176 post-dosing, NBIC, NMBIC, and NM3BIC-treated mice were humanely euthanized, while NM2BIC mice were humanely euthanized on day 365. Upon euthanization, tissues (liver, lung, spleen, lymph nodes, kidney, injection site muscle, gut, and rectum) were collected for drug and prodrug quantitation by UPLC-MS/MS and histopathological assessment.

For PK and BD evaluation in SD rats, male rats (weighing 270–300 gm, aged 8 weeks, purchased from SASCO, Wilmington, MA, USA) were administered a single IM injection of either NBIC or NM2BIC at a dose of 45 mg/kg BIC equivalent in the right caudal muscle (n = 5, SD rats in each group). Blood samples were collected for drug quantification on days 1, 3, 7,14, 21, 28, and then every 2 weeks post-day 28. A total of 3 animals in the NBIC group were lost during blood sampling on days 98, 126, and 194, respectively. One animal in the N2MBIC group was lost during blood sampling on day 168. The rats were humanely euthanized on day 365 for tissue (liver, lung, spleen, lymph nodes, kidney, injection site muscle, gut, and rectum) collection.

### 4.7. PK and BD studies of the dimeric prodrug nanosuspension

For NMXBIC mice PK studies, male BALB/cJ mice (n = 3), matched in weight and age to those used in monomeric prodrugs PK studies, were administered a single IM injection of NMXBIC at a dose of 45 mg/kg BIC equivalent on day 0. Blood samples were collected on days 1, 3, 7, 14, 21, and 28, and then every two weeks until day 365, when the animals were humanely euthanized. SD rats, matched in weight and age to those used in monomeric prodrugs PK studies, were divided into three groups. Group 1 (n = 5 SD rats) was injected with a single dose of 45 mg/kg BIC equivalent NMXBIC_low concentratio_n on day 0. Group 2 (n = 8 SD rats) was injected with a single dose of 45 mg/kg BIC equivalent NMXBIC formulation, while Group 3 (n = 8 SD rats) was injected with a higher dose of 90 mg/kg BIC equivalent NMXBIC on day 0. All the injections were administered by IM into the right caudal muscle. For group 1, blood was collected from all five animals on days 1, 3, 7, 14, 21, 28, and every two weeks until day 365, when animals were humanely euthanized. For groups 2 and 3, blood sampling was alternated between four animals. 4 animals from group 2 and 3 each was sacrificed on day 90, while other 4 animsl from group 2 and 3 was sacrificed on 225 and 365, respectively. Tissues were collected for drug and prodrug quantitation by UPLC-MS/MS and histopathological assessment.

For mice and rat studies in both monomeric and dimeric PK studies, a 28 G × ½” needle was used for injections, and the total injection volume did not exceed 1 μl/ gm weight for SD rats or 1.6 μl/gm weights for BALB/cJ mice. Blood samples were collected in heparinized tubes. Blood sampling was collected via submandibular puncture for BALB/cJ mice and via retro-orbital or tail-vein bleeding from SD rats. Plasma was isolated from blood by centrifugation at 2,000x g for 8 mins and analyzed for drug content by UPLC-MS/MS using methods specified in the supplementary section. All the rodents were euthanized using isoflurane, followed by cervical dislocation. The tissues were homogenized and analyzed for drug content by UPLC-MS/MS. Non-compartmental PK for plasma BIC in all species was performed by Phoenix WinNonlin-8.3.3.33 software (Certara, Princeton, NJ, USA). Safety evaluation in SD rats and BALB/cJ mice was assessed by monitoring animal body weights, organ to body weight ratios after sacrifice, serum chemistry, and tissue histopathology. For tissue histopathology assessments, 5-μM paraffin-embedded tissue sections were stained with hematoxylin and eosin and imaged according to a previously published protocol [46]. An independent certified pathologist assessed the imaged tissue sections in a blinded way. A VetScan VS-2 instrument (Abaxis Veterinary Diagnostics) was used to assess serum chemistry profiles, and the data was compared with age-matched control SD rats or BALB/cJ mice.

All the animal experiments were approved by the UNMC Institutional Animal Care and Use Committee, and animals were regularly monitored by animal care personnel and veterinary staff from the Comparative Medicine department at UNMC. BALB/cJ mice and SD rats used in the PK studies were kept in controlled conditions, including a 12-hour light/dark cycle, temperatures ranging from 20 to 24°C, and humidity levels between 30% and 70%. The animals were given a sterilized 7012 Teklad diet (Harlan, Madison, WI) and had access to acidified water ad libitum.

### 4.8. Injection site reactions in NZW rabbits

The local injection site study was conducted using a protocol approved by the Institutional Animal Care and Use Committee (IACUC) at Labcorp Early Development Laboratories Inc. (Madison, WI, USA-53704). Twenty-four female NZW rabbits were grouped into four (Groups 1–4), each comprising six animals. Each animal in group 1 received a total of four subcutaneous injections in four distinct sites on day 1 and day 26 (one injection of NM2BIC and one injection of placebo each day). On day 1, 600 mg of NM2BIC (2 ml in volume) and placebo (2 ml) were administered subcutaneously in the upper right and upper left scapular regions, respectively. On day 26, a similar dose of NM2BIC and placebo were given subcutaneously in the lower right and lower left scapular regions, respectively.

Each animal in group 2 received a total of four IM injections in four distinct muscle sites on day 1 and day 26 (one IM injection of NM2BIC and one IM injection of placebo each day). On day 1, they received 300 mg of NM2BIC (1 ml in volume) and a placebo (1 ml in volume) in the right thigh and left paralumbar muscles, respectively. On day 26, a second injection of a similar dose of NM2BIC and placebo were administered in the left thigh and right paralumbar muscle, respectively.

Group 3 mirrored the administration patterns of Groups 1, substituting 600 mg of NM2BIC (2 ml) for 300 mg of NMXBIC (2 ml). Similarly, Group 4 mirrored the administration patterns of Group 2, substitution 300 mg of NM2BIC (1 ml) for 150 mg of NMXBIC (1 ml). The vehicle control for every group consisted of 88.6% PBS (pH 7), 5.7% PEG3350, and 5.7% Tween 20.

All the injection sites were shaved and cleaned with a surgical scrub before administration. Dose sites were marked and maintained throughout the study. Dose sites were observed daily, including immediately post-dose on Days 1 and 26 and just before necropsy on Day 29, and scored/graded using a Draize technique. Abnormal observations or indications of ordinary were recorded. If edema or erythema at the injection site was observed, measurements of the affected area were recorded separately (in cm) for each finding accordingly. Photographs of abnormal dose site observations (discharge, ulceration, etc.), if noted, were taken. Animals were anesthetized with sodium pentobarbital and exsanguinated on day 29 post-first injection. Injection sites from each animal were collected and preserved in 10% neutral buffered formalin: Injection Sites (4 sites/ animal), including the overlying skin, stapled to cardboard to maintain the orientation of the dose site and any Gross Lesions (if present). Collected tissues were embedded in paraffin, cut into five μM sections, and stained with stained with hematoxylin and eosin for histopathological assessment. Histology slides were examined by Experimental Pathology Laboratories Inc. (Sterling, VA, USA-20166)

Histology slides were subjected to qualitative assessment based on the Draize scale, which included diagnosis and grading of the overall inflammatory response and cellular infiltrate, local tissue reaction and/or damage, including tissue degeneration/necrosis, grading of edema, hemorrhage, fibrosis, or formation of granulation tissue. The severity of the non-neoplastic tissue lesions was graded on a scale of 1 to 5, represented by minimal, mild, moderate, marked, and severe Non-gradable lesions, such as cysts, were noted as “P” for present. A detailed description of the severity grading scale is included in the supplementary sections (Supplementary Table 3).

### 4.9. Isolation and cultivation of human monocytes.

Monocytes from HIV-1/2 and hepatitis B seronegative donors were acquired through leukapheresis and purified using a counter-current centrifugal elutriation process. Following isolation, the monocytes were cultured according to protocols described in our prior publications [27, 47]. After differentiation, MDM were used to evaluate drug-particle uptake, retention, and antiretroviral activity.

### 4.10. Prodrug nanoformulations uptake and retention in MDMs

MDM uptake and retention of BIC prodrug nanoformulations followed the methodology detailed in our previously published work [27, 47]. In brief, for uptake studies, MDMs were exposed to either 25 μM of BIC nanoformulation (NBIC) or prodrug nanoformulation for 24-hours. In contrast, for retention studies, MDMs were initially treated with 25 μM of NBIC or prodrug nanoformulations for 8 hours and subsequently cultured in a drug-free medium for a duration of 30 days. To determine the intracellular BIC and prodrug levels in both uptake and retention studies samples, MDM samples were collected at specific time intervals, lysed in methanol, and quantified using UV-Vis UPLC, as elaborated in the supplementary sections.

### 4.11. BIC prodrug nanoformulations in HIV-1_ADA_-challenged MDMs

Assessments of the antiretroviral effectiveness of BIC prodrug nanoformulations were performed according to the methodology established in our previously published research [27, 47]. In brief, MDMs were initially exposed to the 25 μM of either NBIC or prodrugs nanoformulation for 8 hours and subsequently cultured in a drug-free medium for 30 days. At specific time intervals, MDMs were exposed to HIV-1 _ADA_ at a multiplicity of infection (MOI) of 0.1 infectious virions per cell for 16 hours, following the protocols outlined in our earlier publications [27, 47]. To perform the HIV-1p24 antigen immunohistochemistry, the MDMs were fixed with 4% PFA at each time point. Furthermore, post-challenge cell culture supernatants were collected to determine the reverse transcriptase (RT) activity percentage in HIV-1 infected cells.

### 4.12. BIC prodrugs half-maximal inhibitory concentration (IC_50_)

A previously published protocol was used to determine the IC_50_ in MDMs [27, 47]. In brief, MDMs were exposed to varying concentrations, ranging from 0.01 to 1000 nM of either BIC or prodrug solution, dissolved in 0.1% DMSO(v/v) or nanoformulations. One hour after the drug or nanoformulation treatment, MDMs were challenged with HIV-1 _ADA_ at an MOI of 0.1 for 16 hours. After that, cells were cultured in drug-containing media. On day 10, cell supernatants were collected and analyzed for HIV-1 RT activity.

### 4.13. Statistics and reproducibility

Microsoft Excel V16.49 (Redmond, WA, USA) and GraphPad Prism 9.3 software (La Jolla, CA, USA) were used for all data analysis and reported as mean ± SEM. Extreme outliers, predefined as values greater than 3-fold of the mean and beyond the 99% confidence interval of the mean, were excluded from the analysis. Chemical synthesis, physiochemical characterization of the synthesized compound, and formulation production were successfully reproduced at least five times. Enzymatic and chemical hydrolysis and all in-vitro experiments were performed using at least three biologically distinct replicates and repeated at least twice with successful replication of the results.

All animal data sets were reproduced in three animal species (mice, rats, and an ongoing rhesus macaque study). Sample sizes were chosen to achieve statistical significance while considering cost and ethical animal use criteria. Although the sample size was not determined through power analysis, it proved adequate to establish statistical significance in all PK experiments. For mice PK studies, the sample size was 3 or 5 per group, while in SD rats studies, the sample size was 5 or 8. Due to experimental constraints, investigators were not blinded during experiments or sample collection but employed an unbiased approach. Nevertheless, all animals were randomly allocated to the experimental groups, and efforts were made to ensure unbiased data through separate roles of investigators in sample and data collection/analysis for animal experiments (including drug quantification, CBC counts, and serum chemistry) and blinded pathological evaluation by the pathologist.

### 4.14. Study approval

The University of Nebraska Medical Center Institutional Animal Care and Use Committee (IACUC) approved all the PK studies involving laboratory animals by the standards incorporated in the Guide for the Care and Use of Laboratory Animals [48]. Human peripheral blood monocyte isolation and purification from HIV-1/2 and HBV seronegative participants were performed according to an excempt protocol by the UNMC Institutional Review Board (IRB) with informed consent.

## Figures and Tables

**Table 1:** Injection site reaction profile in NZW rabbits after two IM injections of 150 mg of NMXBIC.

Time Point		Day-3 Post-second injection		Day-29 Post-first injection	
Treatment		Vehicle	NMXBIC	Vehicle	NMXBIC
Injection site		Right paralumbar muscle	left thigh muscle	Left paralumbar muscle	right thigh muscle
No. of Animals Examined		6	6	6	6
Myofiber necrosis	Incidence	3	1	none	none
	Grade (n)	minimal (1), mild (2)	Minimal (1)		
Intramuscular fibrosis	Incidence	none	none	1	none
	Grade (n)			Minimal (1)	
Intramuscular inflammation and pyogranulomatous	Incidence	none	none	none	1
	Grade (n)				Moderate (1)
Intramuscular mixed cell Inflammation	Incidence	3	6	none	none
	Grade (n)	minimal (3)	minimal (5), mild (1)		
Intramuscular pseudocyst	Incidence	none	none	none	1

* The severity of the tissue lesions is graded according to Supplementary Table 3: Severity grading scale

**Table 2:** Injection site reaction profile in NZW rabbits after two IM injections of 300 mg of NM2BIC.

Time Point		Day-3 Post-second injection		Day-29 Post-first injection	
Treatment		Vehicle	NM2BIC	Vehicle	NM2BIC
Injection site		Right paralumbar muscle	left thigh muscle	Left paralumbar muscle	right thigh muscle
No. of Animals Examined		6	6	6	6
Myofiber necrosis	Incidence	2	1	none	none
	Grade (n)	minimal (2)	Minimal (1)		
Intramuscular inflammation and granulomatous	Incidence	none	none	none	2
	Grade (n)				Moderate (2)
Intramuscular mixed cell Inflammation	Incidence	2	3	none	none
	Grade (n)	Minimal (2)	minimal (2), mild (1)		
Intramuscular pseudocyst	Incidence	none	none	none	2

* The severity of the tissue lesions is graded according to Supplementary Table 3: Severity grading scale
